# The Selfie Paradox: Nobody Seems to Like Them Yet Everyone Has Reasons to Take Them. An Exploration of Psychological Functions of Selfies in Self-Presentation

**DOI:** 10.3389/fpsyg.2017.00007

**Published:** 2017-01-17

**Authors:** Sarah Diefenbach, Lara Christoforakos

**Affiliations:** Department of Psychology, Ludwig-Maximilians-University MunichMunich, Germany

**Keywords:** selfies, self-presentation, motivations, affective experience, self vs. other judgments, selfie bias

## Abstract

Selfies appear as a double-edged phenomenon. Taking, posting, and viewing selfies has become a daily habit for many. At the same time, research revealed that selfies often evoke criticism and disrespect, and are associated with non-authenticity and narcissism. The present study (*N* = 238) sheds further light on the somewhat contradictory phenomenon of selfies and their psychological value. In addition to previous studies on selfies and personality traits, the present research explores relations to popular, habitual self-presentation strategies, self-reflections on own and others’ selfie-taking behavior, selfie-related affect, and perceived consequences of selfies, by applying a combination of self-constructed and existing scales [e.g., habitual self-presentation scales (Merzbacher, 2007), Positive and Negative Affect Schedule (Watson et al., 1988)]. Our findings confirmed habitual self-presentation strategies as a relevant factor for understanding selfies: Participants scoring high on self-promotion (promoting one’s strength and abilities) and self-disclosure (revealing one’s feelings for earning sympathy) felt especially positive while takings selfies, whereas understatement was correlated with negative feelings. Nevertheless, self-presentational motives were rather attributed to others’ selfies than to own selfies. Moreover, others were assumed to have more fun and positive feelings while taking selfies whereas own selfies were judged as more authentic and self-ironic. Altogether, participants expressed a distanced attitude toward selfies, with stronger agreement for potential negative consequences (threats to self-esteem, illusionary world) than for positive consequences (e.g., relatedness, independence), and a clear preference (82%) for viewing more usual pictures instead of selfies in social media. The revealed selfie-bias, i.e., the systematic discrepancy between judgments on own versus others’ selfies, and the reported critical attitude toward selfies allows multiple interpretations. Taking peoples’ statements literally, selfies should have never become as popular as they actually are. On the other hand, the selfie bias may fulfill a psychological function. Perceiving one’s own selfie behavior as self-ironic and only half-committed, allows to fulfill self-presentational needs without feeling narcissistic. In conclusion, we suggest that the playful and somewhat ambiguous support of self-presentation may be a key factor for the success of selfies. Relations to biases and mechanisms from social psychology, limitations of the present study and implications for future research are discussed.

## Introduction

Selfies have become enormously popular and it is nearly impossible to visit any social media site without seeing our friends’ faces in close-up. A selfie is a self-portrait photograph of oneself (or of oneself and other people), taken with a (phone) camera held at arm’s length or pointed at a mirror, that is usually shared through social media (Sorokowski et al., 2015). Though exact data about the worldwide pervasiveness of selfies are not available, the estimations in existing selfie statistics are impressive. For example, the *Google* statistics in 2014 (Brandt, 2014) reported about 93 billion selfies taken per day – counting only *Android* phone users. According to a poll with 3,000 people, among those aged 18–24, every third picture taken is a selfie (Hall, 2013). Selfie accessories, such as selfie-sticks, have been bestsellers, and phone producers have adjusted their products for the sake of selfies. The *Sony Xperia*^TM^ *C3 PROselfie*^TM^ *smartphone, for example*, is equipped with a wide-angle front camera with LED flash and real-time selfie apps. Consequently, in 2013, the term “selfie” was officially added to the Oxford English dictionary, defining a selfie as “a photograph that one has taken of oneself, typically one taken with a smartphone or webcam and shared via social media.” The rising presence of selfies within the last years also becomes visible in language, as Bennett (2014) reports, the usage of the term selfie in English language has raised by 17,000% from 2012 to 2014. In short, taking, posting, and viewing selfies has become a daily habit for many and their mere pervasiveness makes it relevant to know more about the psychology of taking selfies and their consequences on an individual and societal level. The present research aims to contribute to a deeper understanding of selfies through the exploration of related motives and psychological variables, and in particular, the ambivalent character and judgments of selfies.

In fact, the current discussion about the value and consequences of selfies is quite diverse. While some highlight the value of selfies as a new material for creative work and the enhanced possibilities to convey emotions, others are primarily concerned about the excessive self-presentation promoted by selfies, and point at related conflicts, threats to self-esteem or decreased mindfulness. Rettberg (2014), for example, analyses selfies from a cultural perspective. She describes how the selfie culture gives rise to experimentation and mutual inspiration, inventing new genres such as serial selfies, or time-lapse selfies. For instance, the award-winning time-lapse video *Me* by Ahree Lee shows selfies taken every day for 3 years.

In contrast, Roman (2014) focuses on the often negative side-effects of selfies for social interaction. While being totally immersed in the mission of taking the perfect selfie, this may diminish the experience of the moment itself or even cause social conflict. Aiming for the perfect shot of oneself in front of a perfect scenery, people do not seem to care whether they are obstructing the views or disrespecting the needs of others. Selfies, she concludes, “trumped any courtesy, social contract, or even common awareness of the other” (Roman, 2014, p. 314). Another disconcerting phenomenon she sees related to the boom of selfies is the vanishing of natural, candid pictures, and that even young children under 3 years of age are familiar with posing and developing a photo smile. Among adolescents, the enormous focus on taking and sharing pictures of oneself is associated with even more severe effects. For example, sharing selfies among adolescent girls is correlated to overvaluation of shape and weight, body dissatisfaction, as well as thin ideal internalization (McLean et al., 2015), and a high frequency of *Instagram* selfie posting is related to conflict in romantic relationships (Ridgway and Clayton, 2016).

Further reports referred to the relations between selfies and narcissism (Barry et al., 2015; Sorokowski et al., 2015; Weiser, 2015), or the selfie as “a prototype of expressive inauthenticity” (Lobinger and Brantner, 2015). In contrast to “normal,” authentic photographs with natural facial expressions and poses, the participants in the study by Lobinger and Brantner (2015) judged selfies with clearly recognizable poses (e.g., duck face, posing in front of a mirror) as inauthentic way of showing off, often imitating role models from star and celebrity culture rather than showing one’s true self. Another typical element of selfies related to inauthenticity judgments was the visibility of the photographic production process, e.g., selfies in which the arm of the depicted person is visible. Such elements highlight that the depicted person deliberately took this photo, destroying any illusion about a selfie as a natural glimpse into a person’s life. In this sense, a selfie could never show an authentic, natural snapshot of a person’s life. Whatever one was doing, one interrupted this activity to take a selfie. In fact, some self-photographs even play with this aspect and deliberately display inauthenticity, e.g., photos showing a “sleeping” person, but revealing through a mirror that the person has taken the photo^1^^,^^2^. On the other hand, this lacking authenticity may be one reason why people state that they prefer seeing other pictures of their friends than selfies (Christoforakos and Diefenbach, 2016).

Taken together, selfies appear as a somewhat mysterious phenomenon. Aside from art and design projects, the discussed consequences of selfies, seem rather negative – breaking social norms, focusing on photographing oneself rather than what is happening around us, causing conflict in relationships, fostering body dissatisfaction, inauthenticity and narcissistic behavior. Still selfies are extremely popular. They seem to be more for people than just a new trendy way of taking photos. Probably, selfies would not have become so popular if not providing specific value beyond “usual” photos. The present paper illuminates this paradox situation through a psychological perspective and deeper insight into the motivations behind selfies. Our research explores how people may benefit from selfies, how they reflect on selfies and see their own position within the selfie culture, and why selfies could be more prevalent than individual statements suggest.

In the following, we first discuss the theoretical background and considerations behind our work, namely, the possible advantages and value that selfies may provide to people, with a focus on self-presentation and impression management. We also discuss first findings on self-reflection on selfies and differences between self and other judgments. We then present an empirical study that explores these phenomena in more detail, followed by a general discussion and implications for future research.

## Background

### The Potential Value of Selfies – From Self-Exploration to Self-Presentation

At first, and apart from a social dimension, self-portraiture and selfies may be seen as a means for self and identity exploration. Rutledge (2013) highlights the function of selfies as a trigger for self-study and self-observation, supporting our need to “figure out who we are and what we are … whether you are trying to find greater consciousness or figure out what moved you to buy the blue shoes. … we can look back on our motives and actions and gain insight we couldn’t get in any other way.” This inward perspective, however, seems only a small part of the picture. In general, the outward orientation and public presentation seems an essential part of selfies, considering that most people do not take selfies just for themselves. More often, the envisioned audience seems already present while taking the selfie, and people deliberately use self-photographs to form a particular impression. Lyu (2016), for example, explored impression management in the context of travel selfies shared via social networks, revealing how tourists strategically adjust photographic images to manage their impressions and highlighting the role of posting selfies as strategic self-presentation behavior. In line with this, existing definitions of selfies in research (Sorokowski et al., 2015) or the Oxford English dictionary, explicitly mention that selfies are usually shared via social media, or describe selfies as “the posting of self-photographs” (Barry et al., 2015).

In order to better understand the value of selfies as a form of online self-presentation, previous research on social media offers a helpful starting points, especially since sharing photos has become a key feature in social networks (Weiser, 2015). For example, studies regarding the example of *Facebook*, already examined the benefits for identity construction and implicit identity claims through one’s profile photo and other pictures (Zhao et al., 2008), the use of self-promotional content features and its relation to narcissism and self-esteem (Mehdizadeh, 2010), the benefit of online social technologies for identity experimentation and self-disclosure (Best et al., 2014), as well as the challenges of managing multiple self-presentations via different services and profiles (Brivio and Ibarra, 2009). Another strand of research explored relations to self-esteem and well-being. Here, studies showed a positive effect of selfies on self-esteem through the possibilities for selective self-presentation in social media, as for example, editing or examining one’s own *Facebook* profile (Gonzales and Hancock, 2011; Toma, 2013). Visiting the *Facebook* profiles of others, however, can have rather negative impact on well-being, especially if *Facebook* friends are not personally known: while neglecting that this selective view does not represent the “true life” of others, one comes to the depressing conclusion that others must be happier and having better lives (Chou and Edge, 2012). Thus, the same effect that boost our self-esteem when pimping our own profile and presenting a highly selective, favorable insight in our life, may fire back when visiting the profiles of others.

In general, online-self presentation via social media profiles, blog posts, etc., is much more controlled than self-presentation in oﬄine environments, since the former can be edited and revised before making it public, with lots of opportunities to manage the image perceived by others (Stǎnculescu, 2011). Within this, selfies push the opportunities for managing others’ view of oneself to the limit and provide some degree of new independence and control. One can get a quick picture of oneself, anywhere, at any place, without help from others. While taking a photo of oneself via camera held at arm’s length was already possible before the age of smartphones, smartphones and specialized selfie-equipment have brought this form of self-photography to perfection. One not only selects particular pictures for self-presentation but also already starts the ‘management’ process in the very moment of snapshotting one’s life. With the selfie-cam, acting as a mirror, the over controlled self-presentation in social media already starts while taking a photo.

Investigations in relation to individual differences in strategic self-presentation behavior lent further support to self-presentation as a central motive for social media use. Błachnio et al. (2016) explored relations between individual tendencies for different self-presentation styles (e.g., self-promotion, self-depreciation) and *Facebook* usage and found a positive correlation to the individual tendency for self-promotion. Thinking about the specific value of selfies, relations between the individual engagement in taking and posting selfies and individual self-presentation strategies are conceivable as well, as discussed in the following paragraphs.

### Selfies in the Light of Habitual Self-Presentation Strategies

Among the many opportunities of social media, selfies appear as an element with an especially high potential for self-presentation and impression management: *Per se*, selfies put the focus on the self. The selfie cam provides control while taking the picture; photo editing does the rest. With the person’s face in the foreground, selfies can be very expressive pictures, convey emotions and an image as desired. Altogether, selfies thus seem to provide best opportunities for strategic self-presentations and impression management.

However, selfies may be especially supportive of particular types of self-presentation. Given that people vary in their habitual use of different strategies of self-presentation, the enthusiasm for selfies may also vary with how well selfies as a means for self-presentation fit with individually preferred self-presentation strategies. For example, in the taxonomy of self-presentation strategies suggested by Merzbacher (2007), two strategies in particular seem well in line with what selfies can provide: The first strategy is self-promotion, i.e., highlighting own accomplishments and abilities, to be perceived as capable, intelligent, or talented by others (cf. also Jones and Pittman, 1982; Tedeschi and Norman, 1985). By showing a highly controlled picture of oneself in the way that one wants to be seen by others, selfies provide a ground for self-promotion. The second strategy is self-disclosure, i.e., revealing (selective parts of) one’s self and emotions with the aim to convey a likable image and earn sympathy, trust and appreciation from others (cf. also Schlenker, 1980; Tedeschi et al., 1985). In line with this, selfies, “snapshots” of one’s life, offer a lightweight possibility to express emotions and revealing insights into one’s life. Selfies form a “visual diary,” and a way to share emotions with friends and family (Wortham, 2013). In contrast to self-promotion, self-disclosure does not aim to present the best “polished” self, but rather aims for sympathy through openness and “natural” insights into the self (though still being selective insights). Selfie-trends such as the “ugly” selfie or “post shower selfies” may fall into this category.

Other strategies of self-presentation in the taxonomy by (Merzbacher, 2007) seem less compatible with selfies, as for example understatement. Understatement in the sense of a strategic way of self-presentation refers to ostensibly downplaying one’s own relevance, abilities and achievements, but implicitly expecting objection from others, finally leading to a positive revaluation of the self. Selfies, however, seem not well compatible with this strategy. Posting any photo of oneself is already some sign of taking oneself seriously. Posting a selfie, i.e., a photo putting the person in the center seems everything but understatement. Moreover, selfies have no implemented feedback channel as required for effectively using understatement as a strategy with positive effect for the self. An important element of understatement as a self-presentation strategy is the interaction partner who will disclaim the modest self-presentation. Hence, people who habitually use understatement should be less enthusiastic about selfies as a tool for self-presentation.

In sum, opportunities for self-presentation may be assumed as a core attractor for the popularity of selfies. However, selfies may not foster all types of different self-presentation strategies in equal degree, so that the enthusiasm for selfies may vary with individual tendencies in habitual self-presentation.

### Self-reflection on Selfies

From an analytical point of view, self-presentation may be one of the most prominent psychological reasons for taking selfies. However, another interesting question is how people reflect on this issue themselves: Do they see selfies primarily as a tool for self-presentation? Where do they see advantages and disadvantages of selfies in their daily life? How do they reflect on their own and others’ selfie taking behavior?

So far, only little research has explored personal reflections and subjective motivations for taking and posting selfies. An exception is the study by Sung et al. (2016), which explored motivations for posting selfies by an online-survey and a prior interview study. The interview study revealed four primary motives, namely attention seeking, communication, entertainment, and archiving, which each were assessed by a 3–6 items in the online-survey. Among the four motive scales, attention seeking (sample items: “To show off,” “To be acknowledged by others”) seems to have the highest overlap with self-presentation. While the motives attention seeking, communication, and entertainment were positively related to narcissism and selfie-posting frequency, archiving was not.

In an own qualitative study (*N* = 86, see also Christoforakos and Diefenbach, 2016), we explored peoples’ subjective associations with selfies, thereby distinguishing between perceived positive and negative aspects of selfies. Both aspects were surveyed by an open question format and categorized by qualitative content analysis. Overall, the most common positive associations were independence (taking self-portrait pictures without help from others), meaning/documentation (selfies as a marker of meaning, selfies as memories), relatedness (feeling close to people when seeing their selfies), control/self-staging (control over the picture and the image perceived by others), and positive feelings (e.g., fun, chasing boredom). In contrast, as the most negative consequences of selfies participants named illusion/fake (inauthentic, unnatural pictures, creating a superficial illusionary world), threat to self-esteem (e.g., risking negative reactions from others, vulnerability), negative impression on others (e.g., narcissistic, showy), bad quality pictures, and unnecessary/uninteresting pictures. Hence, our findings on positive associations generally show parallels with the study on selfie motivations by Sung et al. (2016), e.g., relatedness – communication, meaning/documentation – archiving, positive feelings – entertainment. However, the aspect of control and self-staging was brought up more explicitly in our study, and also the aspect of independence as a positive consequence of selfies was not discussed by Sung et al. (2016).

Moreover, an interesting tendency in our qualitative data (Christoforakos and Diefenbach, 2016) was a different form of argumentation when talking about one’s own selfie habits (e.g., “for me, it is a form of documentation”) versus others taking selfies and general judgments (e.g., “the people get more narcissistic”). Not all statements were clearly indicative of self versus other judgments, since the study did not explicitly ask for this differentiation. However, those statements that did, showed a focus on situational and practical reasons for taking selfies oneself (e.g., “a quick photo without needing help from others,” “using the selfie-cam as a mirror”) whereas other judgments rather referred to reasons lying in the person (e.g., self-admiring, narcissistic), depicting the prototypical selfie-taker as the “type of character who needs it.” We took this, as a hint for a more systematic exploration of judgments for own selfies versus others ‘selfies and peoples’ reflections on selfies as a societal phenomenon. In general, the exploration of interpretations and attributed reasons for taking selfies can offer deeper insight into the psychology and subjective experience of selfies.

### Aims of the Study

The present empirical study followed several aims:

First, an exploration of psychological functions of selfies with a special focus on selfies as a means of self-presentation as well as the representation of common self-presentation strategies. We focused on the strategies of self-promotion, self-disclosure and understatement, assuming positive relations with selfie-related affect for the two former and negative relations for the latter.

Second, an exploration of the image and perceived consequences of selfies, and relations to personal and societal values. Thus, besides indirect conclusions about the value of selfies (e.g., correlations between selfie-related affect and habitual self-presentation strategies), our study also surveyed explicit reflections about how people perceived selfies and their consequences in our social interaction.

Third, based on the incidences for differences between self- versus other judgments in our previous research (Christoforakos and Diefenbach, 2016), we aimed for a systematic exploration of this effect. In line with a self-serving interpretation, we assumed more likable judgments (e.g., self-ironic) for own selfies, and a more critical view (e.g., non-authentic) of others’ selfies.

## Materials and Methods

### Participants

Two hundred thirty-eight individuals (167 female) living in Germany, Austria, and Switzerland took part in the study and completed the whole survey. The age range was between 18 and 63 years (*M* = 25.33; *SD* = 7.21).

### Procedure

The study was carried out via online survey with unipark ^3^, and participation took about 15 min. All materials were presented in German language. An invitation link to the study was distributed via diverse mailings lists and university panels. As an incentive, three Amazon gift vouchers (50€) were raﬄed among all participants who completed the survey. Participants’ selfie behavior and related variables were assessed by a number of measures, as listed in the next sections.

### Measures

#### Selfie Behavior and Preferences

Participants indicated how often they were usually taking selfies and receiving selfies from friends. Both measures were assessed on a 6-point scale (1 = never, 2 = once a month, 3 = once a week, 4 = several times a week, 5 = once a day, 6 = several times a day). In addition, participants rated how much they liked seeing selfies compared to usual (non-selfie) pictures. Preferences were assessed by a 5-point scale (1 = I prefer selfies, 5 = I prefer usual pictures).

#### Selfie-Related Affect

Participants described their emotional experience when taking selfies with the Positive and Negative Affect Schedule (PANAS; Watson et al., 1988) in German translation by Krohne et al. (1996). Its short form (Mackinnon et al., 1999) consists of five items assessing positive affect (PA, e.g., enthusiastic, inspired) and five items assessing negative affect (NA, e.g., enthusiastic, inspired). The 10 items were presented in random order. Judgments were assessed on a 5-point scale (1 = not at all, 2 = a little, 3 = moderately, 4 = quite a bit, 5 = extremely) and scale values calculated by averaging the according items. Cronbach’s Alpha was 0.80 for PA and 0.68 for NA. Despite the low scale reliability for NA, we left the scale in original form to facilitate comparison with previous studies.

#### Self-Presentation Strategies

Individual self-presentation strategies were assessed by a selection of items from the habitual self-presentation scales by Merzbacher (2007), who built on the self-presentation tactics scale by Lee et al. (1999). We focused on those facets of self-presentation, which we assumed as particularly relevant in the context of selfies, i.e., self-promotion and self-disclosure. We further assessed understatement, assuming that this strategy is *not* supported through selfies, thus being able to check a potential differential effect. Each strategy (self-promotion, self-disclosure, understatement) was assessed with five statements, e.g., “I tell others about my successes” (self-promotion), “I show my feelings to be well received by others” (self-disclosure), “I deliberately downplay my achievements” (understatement). The 15 statements were presented in random order. Participants judged how well the different statements described their typical behavior on a 9-point scale (1 = never, 9 = most of the time). Scale values were calculated by averaging the according items with satisfying scale reliability (Cronbach’s Alphas: self-promotion 0.84, self-disclosure 0.78, understatement 0.78). A principal component analysis (varimax rotation, 58% explained variance) with three components to be extracted revealed a satisfactory solution with the five items assessing one strategy forming one component, and no loadings larger than 0.30 on other components.

#### Perceived Consequences of Selfies

Perceived consequences of selfies were assessed based on a previous qualitative study, where we surveyed most prominent positive and negative associations related to selfies (as mentioned in the Background section, see also Christoforakos and Diefenbach, 2016). For the present study, we focused on six aspects, four of them being named as positive effects of selfies (independence, meaning, relatedness, self-staging) and two of them being named as negative effects of selfies (illusionary world, threat to self-esteem). Each aspect was assessed with two items presented in random order. Sample items are “Selfies provide independence” (independence), “Selfies provide opportunities to feel close to others” (relatedness), or “Selfies show an illusionary world” (illusionary world). Participants indicated their agreement on a 5-point scale (1 = not at all agree, 5 = totally agree). Scale values were calculated by averaging the according items, with scale reliabilities between 0.62 and 0.79. A principal component analysis (varimax rotation, 79% explained variance) with six components to be extracted revealed a satisfactory solution with the two items assessing one aspect forming one component, and no loadings larger than 0.30 on other components.

#### Statements on Own versus Others’ Selfies

Judgments on own selfies and others’ selfies was assessed with 10 statements, relating to five different aspects: self-irony (“My/Other peoples’ selfies are often funny or self-ironic”), authenticity (“My/Other peoples’ selfies show my/their true personality”), self-presentation (“I/Other people use selfies as a means for self-presentation”), fun (“I/Other people take selfies because it is fun”), situational variability (“My/Other peoples’ selfies are very different from one situation to another”). The 10 statements were presented in random order, so that the contrast of judgments on own versus others’ selfies may not have been obvious to participants. For each statement, participants indicated their agreement on a 5-point scale (1 = not at all agree, 5 = totally agree).

## Results and Discussion

### Selfie Behavior and Preferences

Reports on selfie behavior showed a wide range, **Table 1** shows reported frequencies of taking and receiving selfies. For example, 50% declared to take selfies about once a month. A total of 27% stated taking selfies once a week or more often, one participant even several times a day. Statistics for receiving selfies were generally higher, here altogether 49% claimed receiving a selfie at least once a week, and six participants even several times a day. Thus, one seems to receive selfies more often than taking them. Moreover, taking and receiving selfies are positively correlated (non-parametric Spearman correlation ρ = 0.56, *p* < 0.001.), so that the two activities may be interpreted as a general indicator of selfie engagement. The present high variability in self-reported selfie engagement is in line with previous research using objective counts, and also reporting wide ranges and high standard deviations in selfie statistics (Barry et al., 2015; Sorokowski et al., 2015).

**Table 1:** Reported frequencies of taking and receiving selfies.

Selfie behavior frequencies	Never	Once a month	Once a week	Several times a week	Once a day	Several times a day
Taking selfies	22,7%	50%	18,5%	7,6%	0,8%	0,4%
Receiving selfies	12,6%	38,7%	23,1%	20,2%	2,9%	2,5%

The preference rating for selfies versus non-selfie pictures showed a clear preference for more non-selfie pictures. The mean value on the 5-point scale (1 = I prefer selfies, 5 = I prefer usual pictures) was *M* = 4.30 (*SD* = 0.83), significantly deviating from the scale midpoint [*t*(237) = 24.14, *p* < 0.001]. Eighty-two percentage gave a 4 or 5 rating, indicating they would like to view more usual pictures instead of selfies in social media. Though one’s own selfie engagement was correlated to a higher acceptance of selfies (receiving selfies: non-parametric Spearman correlation ρ = -0.22, *p* < 0.001; taking selfies: ρ = -0.30, *p* < 0.001), also within the sub-group of those with high selfie engagement, the wish for more usual pictures was still dominant. Even among those taking selfies themselves once a week or more often (*n* = 65), the preference for more usual photos instead of selfies was still significant [*M* = 3.94, *SD* = 0.085, *t*(64) = 8.95, *p* < 0.001]. The same applied to the subgroup of those receiving selfies once a week or more often [*n* = 116, *M* = 4.16, *SD* = 0.86, *t*(115) = 14.54, *p* < 0.001]. Thus, also people taking many selfies themselves tend not to like viewing others’ selfie-pictures and rather wish for a higher number of usual photos. As a first result, this expresses a somewhat paradox situation, where many people are engaged in selfies, but at the same time wish for a reduction of selfies in social media in favor of more non-selfie pictures, expressing a somewhat distanced attitude toward the value of selfies.

### Selfie-Related Affect and Self-Presentation Strategies

The analysis of participants’ reported emotional experience while taking selfies showed mean values in the lower scale range for both positive affect (*M* = 2.64, *SD* = 0.83) and negative affect (*M* = 1.40, *SD* = 0.49). Yet, positive affect was significantly more pronounced than negative affect [*t*(237) = 21.41, *p* < 0.001], indicating that, on average, taking selfies is an overall rather positive experience. Selfie-related positive affect was also related to selfie engagement, i.e., positively related to the frequency of taking selfies (non-parametric Spearman correlation ρ = 0.32, *p* < 0.01) and receiving selfies (non-parametric Spearman correlation ρ = 0.18, *p* < 0.01).

A further analysis revealed that the experienced positivity of taking selfies differed depending on individually preferred self-presentation strategies: An analysis of variance showed general differences between the specification of the three surveyed self-presentation strategies [*F*(2) = 28.73, *p* < 0.001]. Understatement seems to be the least popular (*M* = 4.03; *SD* = 1.46), whereas self-promotion is more popular (*M* = 4.34; *SD* = 1.38) and self-disclosure most pronounced (*M* = 4.93; *SD* = 1.36). As shown in **Table 2**, high values for self-promotion and self-disclosure were correlated with a positive experience of taking selfies but high values for understatement were correlated with a negative experience of taking selfies.

**Table 2:** Correlations between individual self-presentation strategies and selfie-related affect.

	Individual self-presentation strategies		
Selfie-related affect	Self-promotion	Self-disclosure	Understatement
Positive affect	0.16^∗^	0.19^∗∗^	-0.02
Negative affect	-0.02	0.01	0.33^∗∗^

*^∗^p < 0.05, ^∗∗^*p* < 0.01*.

A likely interpretation is that people who tend to understate their successes and competencies when presenting themselves cannot profit from selfies – at least not as a means for self-presentation – and thus associate negative emotions with taking selfies. Taking a selfie, inevitably claiming attention for oneself, is contradictory to such habits of self-presentation. However, for many others, making use of the more popular strategies of self-promotion and self-disclosure, selfies form a suitable means for self-presentation in line with their preferences and, thus, are associated with positive emotions. As discussed above, selfies seem to be a good possibility for selective self-presentation with a focus on strengths, accomplishments, and abilities (self-promotion) as well as displaying emotions, likable openness and insights into one’s life (self-disclosure).

In sum, the pattern of correlations suggests that the self-presentation perspective is crucial for understanding the value of selfies. Also, the consideration of habitual self-presentation strategies helps to explain individual differences in selfie-related affect and liking. In line with our expectations, self-promotion and self-disclosure were related to positive selfie-related affect and understatement to negative selfie-related affect. In other words, people who habitually use self-promotion and/or self-disclosure as strategies of self-presentation also appeared as the most passionate about selfies.

The idea of a relation between taking selfies and self-promotion is quite parallel to previous research on using selfies for impression management, such as strategically adjusted travel selfies (Lyu, 2016), or self-promotion as a major driver of Facebook use (Carpenter, 2012; Błachnio et al., 2016). Moreover, also the wide strand of research exploring relations to narcissism already discussed the potential self-promotional aspects of selfies. For example, Barry et al. (2015, p. 3) described selfies as “inherently self focused [photos], with some perhaps being blatant attempts to gain attention from others due to one’s appearance, affiliations, or accomplishments.” (Sorokowski et al., 2015) explored different sub facets of narcissism and revealed admiration demand as the most important predictor of selfie-posting behavior, in fact, the only narcissism subscale that significantly predicted selfie-posting among women.

The relation between posting selfies and self-disclosure as a self-presentation strategy has, to our knowledge, not been addressed empirically so far. Anecdotic reports already highlighted the potential of selfies for expressing and communicating emotions to others, e.g., “it is about showing your friends and family your elation when you’re having a good day or opening a dialog or line of communication using an image the same way you might simply text ‘hi’ or ‘what’s up?”’ (Wortham, 2013). Our research, however, suggests that self-disclosure through selfies may also fulfill functions beyond opening a line of communication, namely, self-disclosure to earn sympathy, in the sense of strategic self-presentation (Merzbacher, 2007).

Taken together, selfies appear as a powerful tool for impression management, i.e., “the process by which people control the impressions others form of them” (Leary and Kowalski, 1990). However, the usefulness of that tool depends on individually preferred strategies of self-presentation. While self-promotion and self-disclosure are well supported, understatement and possibly also other strategies (which we did not assess in the present study) are not supported. In consequence, people preferring understatement rather show an antipathy for selfies and report negative affect while taking selfies.

### Perceived Consequences of Selfies

Mean values of agreement for the studied perceived consequences of selfies are given in **Table 3**. The analysis showed significant agreement for the potential negative effects of selfies (illusionary world, threat to self-esteem) but only partial agreement for the potential positive effects. Among the potential positive effects, the only aspect that reached significant agreement was self-staging, i.e., the possibility to use selfies for presenting an intended image to others. However, fewer participants acknowledged positive effects of selfies regarding independence, meaning, or relatedness, and mean values of agreement remained significantly below the neutral scale midpoint. In fact, only a small part of the sample showed agreement for positive aspects and scored above the scale midpoint (independence: 14%, meaning: 14%, relatedness: 8%), whereas the ratio of participants scoring above the scale midpoint was 62% for self-staging, 62% for threat to self-esteem, and 67% for illusionary world.

**Table 3:** Mean values of agreement and significance of deviation from scale midpoint (=3) for perceived consequences of selfies.

Perceived consequences of selfies	*M*	*SD*	*t*	*df*	*p*
Positive					
Independence	2.03	0.94	16.02	237	<0.001
Meaning	2.20	1.02	12.07	237	<0.001
Relatedness	1.99	0.85	18.34	237	<0.001
Self-staging	3.50	1.01	5.51	237	<0.001
Negative					
Illusionary world	3.63	1.01	9.66	237	<0.001
Threat to self-esteem	4.49	0.94	7.96	237	<0.001

An analysis of correlations between perceived consequences of selfies and selfie-related affect as well as selfie behavior (see **Table 4**) showed a plausible pattern: those who frequently take selfies themselves reported higher agreement for the positive and lower agreement for the negative consequences of selfies. Also, agreement for the positive consequences of selfies was related to more positive selfie-related affect, and agreement for the negative consequences of selfies was related to more negative selfie-related affect.

**Table 4:** Correlations between perceived consequences of selfies, selfie behavior, and selfie-related affect.

Perceived consequences of selfies	Taking selfies	Selfie-related positive affect	Selfie-related negative affect
Positive			
Independence	0.34^∗∗^	0.39^∗∗^	0.16^∗^
Meaning	0.22^∗∗^	0.29^∗∗^	0.14^∗^
Relatedness	0.18^∗∗^	0.23^∗∗^	0.21^∗∗^
Self-staging	0.20^∗∗^	0.25^∗∗^	0.07
Negative			
Illusionary world	-0.28^∗∗^	-0.05	0.17^∗^
Threat to self-esteem	0.07	0.08	0.14^∗^

*^∗^p < 0.05, ^∗∗^*p* < 0.01*.

In sum, those frequently taking selfies and feeling good while doing so are also more optimistic about the general consequences of selfies. However, according to our results, the majority of participants sees the most obvious consequences of selfies on the negative side, i.e., threat to self-esteem and creating an illusionary world. This parallels previous research, discussing the potential danger of selfies for one’s confidence and self-esteem, emerging from repeated attempts to achieve the “perfect selfie” and the absence of positive feedback (Barry et al., 2015).

On the positive side, the most dominant aspect is self-staging. Other positive aspects such as feelings of relatedness, autonomy or meaning were only experienced by a small part of the participants. Those also appeared as most passionate about selfies, frequently taking selfies and feeling good while doing so. In a way, taking selfies may be a self-intensifying process, where one discovers unexpected positive aspects (besides self-staging) while engaging in the activity and this positive experience encourages further engagement. Nevertheless, the majority showed a rather critical attitude, and among the perceived consequences of selfies, negative aspects clearly predominate. If selfies are good for anything, it is self-staging, at least in the majority’s opinion.

**FIGURE 1:**
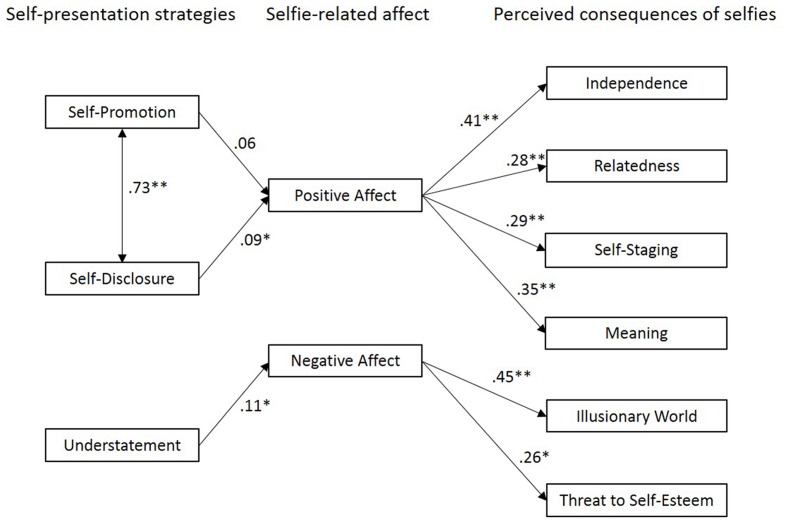
**Path analysis of relationships between self-presentation strategies, selfie-related affect, and perceived consequences of selfies**. Significant pathways are indicated with an asterisk, ^∗^*p* < 0.05, ^∗∗^*p* < 0.01. Residuals are not shown to simplify presentation.

For a comprehensive picture of the relationships between participants’ individually preferred self-presentation strategies, selfie-related affect, and perceived consequences of selfies, we conducted a *post hoc* path analysis computed with R package lavaan. Considering self-presentation strategies as exogenous person variable and based on the found correlational patterns, the tested model assumed effects of self-presentation strategies on selfie-related affect, and, in turn, effects of selfie-related affect on perceived consequences of selfies (**Figure 1**). The fit indices indicated a good model fit, CFI = 0.957; RMSEA = 0.046; SRMR = 0.067 (McDonald and Ho, 2002; Beauducel and Wittmann, 2005; Kline, 2015). The χ^2^ test is significant [χ^2^(30) = 44.963; *p* = 0.039], yet this is a usual consequence of the high number of participants (Bühner, 2011). In sum, individual self-presentation strategies seem to be deciding whether one experiences taking selfies as positive or negative, and the resulting valence of affect implies a focus on either positive or negative consequences of selfies. While positive selfie-related affect goes along with positive judgments on selfies, highlighting consequences such as relatedness, autonomy, meaning, and self-staging, negative selfie-related affect implies agreement to negative consequences of selfies such as creating an illusionary world and threats to self-esteem. Altogether, the present *post hoc* model, suggesting a path from person variables (self-presentation strategies) via affective consequences of selfies to cognitive judgments (i.e., perceived consequences), provides a plausible structure for our data and could be used for further research.

### Statements on Own versus Others’ Selfies

**Table 5** shows mean values of agreement to statements on own versus others’ selfies. Significant differences between own versus other statements occurred for all studied aspects, namely, self-irony, authenticity, self-presentation, fun, and situational variability. Altogether, the findings confirmed our expectations, showing a more likable interpretation of own selfies and a more critical interpretation of others’ selfies: Own selfies were rated as more self-ironic and thought of as more authentic than those of others. In contrast, others were assumed to use selfies for self-presentation and have fun while taking selfies to a higher degree than oneself. A further analysis of the ratios of agreement (i.e., number of 4 or 5 ratings) showed the discrepancy between own versus other statements in more detail. For example, 40% claimed self-irony for their own selfies, but only 13% perceived self-irony in others’ selfies. In contrast, 90% declared others’ selfies as means of self-presentation, but only 46% attested this to own selfies. Obviously, there is a systematic discrepancy in the perception between own versus others’ selfies, i.e., a selfie bias.

**Table 5:** Mean values of agreement and significance of differences for statements on own versus others’ selfies.

	Agreement own selfies		Agreement others’ selfies				
Statement	*M*	*SD*	*M*	*SD*	*t*	*df*	*p*
Self-irony: My/Other peoples’ selfies are often funny or self-ironic.	3.08	1.25	2.58	0.84	5.71	237	<0.001
Authenticity: My/Other peoples’ selfies show my/their true personality.	2.50	1.05	1.88	0.80	8.12	237	<0.001
Self-presentation: I/Other people use selfies as a means for self-presentation.	3.06	1.35	4.38	0.75	14.10	237	<0.001
Fun: I/Other people take selfies because it is fun.	3.10	1.31	3.97	0.82	9.84	237	<0.001
Situational variability: My/Other peoples’ selfies are very different from one situation to another.	3.28	1.24	3.61	1.07	3.88	237	<0.001

A non-expected result was that others’ selfies were assigned a higher degree of situational variability, e.g., showing different images or poses from one situation to another. In consideration of our previous study (Christoforakos and Diefenbach, 2016), where participants focused on situational and practical reasons for taking selfies oneself but personal factors for others’ selfies, we had expected that participants would disregard variations in others’ selfies between different situations. This, however, was not the case. Though the agreement for individual aspects (e.g., self-presentational needs, having fun) was indeed higher, people also acknowledged situational variations in others’ selfies. A problem in our operationalization might be, however, that our items assessing situational variability only asked for observable variations and not to what degree the situation (in contrast to character) influenced the behavior. One could still imagine that the trigger for taking a selfie lies in the person (self-presentational needs), and the situation is rather used to justify a selfie, and adjusting the pose to the surrounding. In this sense, the lower ratings for situational variability for oneself compared to others may also be a statement that oneself is not taking part in the game. Apart from that, it is plausible, that, along with people’s general need for personal control and influence (Frey and Jonas, 2002; Pittman and Zeigler, 2007), one might not want to state situational factors more responsible for one’s actions than internal, personal factors.

In summary, peoples’ statements on own versus others’ selfies suggest a distanced attitude toward taking selfies. In an extreme interpretation, takings selfies may appear below one’s standards. It occurs as a superficial activity, good for others to have fun and realize their needs for self-presentation, but oneself does not take the passion for selfies too seriously. While “the others” appear as the real selfie-takers, this does not mean one totally refuses engagement in selfies. However, if one takes selfies, these are not the typical ones but more authentic or more self-ironic than those of others. While it is possible, that people have actually difficulties in understanding each other’s sense of humor, and can hardly detect signs of self-irony in others’ selfies, this pattern is also in line with a self-serving bias and social demand effects. Self-presentation is the dominant impression of others’ selfies, but for oneself, more favorable motives are constructed. The explicit reflection on one’s selfie behavior, and realizing participation in an activity that one essentially sees as ridiculous, may also be a classic case of cognitive dissonance through a realized gap between attitude and behavior (Festinger, 1957). This dissonance may be reduced by downplaying the narcissistic parts of it and justifying selfie-taking with self-irony or authentic insights into one’s life. Altogether, the present patterns of findings suggests a somehow biased view and romanticization of one’s own selfie behavior. However, several mechanisms may play a role and in the present study, and effects of true misperceptions (e.g., not seeing the irony in others’ selfies, really seeing own selfies as more authentic) and needs for internal and external justification cannot be separated entirely.

## General Discussion

The present study provided advanced insights into the psychological motivations and perceived benefits of taking selfies, with a particular focus on self-presentational aspects as well as peoples’ reflections on selfies and their consequences on an individual and societal level. In addition to previous research, that explored relations between selfie engagement and personality traits (Barry et al., 2015; Sorokowski et al., 2015; Weiser, 2015), the present study highlighted relations to popular, habitual self-presentation strategies. First previous studies on self-reported motivations to take selfies (Sung et al., 2016) have been advanced by a broader study of perceived consequences and insights into peoples’ self-reflections on their own and others’ selfie-taking behavior. Our findings confirmed self-presentation as relevant for the popularity and attractiveness of selfies, but also revealed that this kind of attractiveness is hardly reflected in explicit commitment to selfies. A consideration in light of biases and mechanisms described in social psychology may help to understand this seeming contradictory, or, the selfie bias or selfie-paradox. In the following, we summarize our study findings and then discuss alternative interpretations, parallels to selected mechanisms from social psychology and self-presentation research, and following research questions.

In summary, our findings outline selfies as a complex and somewhat conflicting practice, with less general agreement than the wide dissemination of selfies in social media may suggest. Participants’ reports on their own selfie-taking behavior showed that a considerable part of participants was regularly taking selfies, however, with different levels of positive affect related to it. Further analysis revealed that the experienced positivity while taking selfies differed depending on individually preferred self-presentation strategies. In line with our expectations, particularly participants who habitually use self-promotion and/or self-disclosure as strategies of self-presentation appeared as the most passionate about selfies. For them, selfies may form a welcome opportunity for supporting their naturally preferred self-presentational behavior. In line with this, the most agreed benefit of selfies was self-staging (62%). Other positive aspects such as independence, meaning, and relatedness (which a prior study had revealed as potential positive consequences of selfies), received lower agreement, and were only acknowledged by small parts of the sample (8–14%). In contrast, a much higher part of participants (62–67%) declared agreement for potential negative consequences, such as selfies creating an illusionary world and threats to self-esteem. This overall rather negative view on selfies was continued with the finding that the vast majority (82%) declared they would like to see more usual pictures instead of selfies in social media. Thus, though (occasionally) being part of the selfie culture themselves, there is also a sense of reflection that more non-selfie pictures could be desirable.

Such reports suggest that people predominantly perceive negative consequences of selfies, and more selfies are taken than the viewers appreciate. Nevertheless, worldwide people take thousands of selfies each day. Moreover, there are systematic differences in perceptions for one’s own and others’ selfie pictures. As hypothesized, people rated others to have more fun while taking selfies, and assumed a higher relevance of self-presentation through selfies for others than for oneself. Moreover, others’ selfies were rated as less authentic than own selfies, whereas own selfies were assigned a higher degree of self-irony. Though declaring a general wish for less selfies in social media, the single individual seems to find good reasons to take/post selfies from time to time, and interprets own selfies in a way, that make them appear as more justified (authentic, self-ironic) than those of others.

While the present study once more confirmed the self-presentational value of selfies, it seems that understanding their potential for self-presentation is only part of the story of understanding selfies. The even more interesting part is the story that people construct around selfies: The overall critical attitude toward selfies, and wishes for more non-selfie pictures in social media, even among active selfie-takers. When asking the single person, selfies should have never become so popular. Taken together, the above described discrepancy between judgments on own versus others’ selfies, the controversial role of self-presentation, and the engagement in an activity that one describes as mainly critical, forms what we denoted as selfie bias, resulting in a paradox: nobody seems to like selfies, yet everyone has reasons to take them.

In a provocative interpretation, the whole sum of selfies may be “exceptional pictures” from people who actually are no fans of selfies. They may just half-heartedly follow the social norms, not wanting to destroy the fun for others. Without taking it seriously or really having a passion for it themselves, they might rather experience selfies as a kind of social obligation, which they secretly hope to stop being popular. If, however, everybody thinks like this yet does not act on it, the observable result is that everybody will further engage in selfies and further contribute to their popularity. This would mean having a mass of people establishing a culture that only few seem fully committed to. In this case, a possible implication could be needing to find ways to free people from taking selfies, since it essentially is an activity that only few can profit from and many see as negative.

An alternative line of interpretation could be that many people actually enjoy taking selfies and profit from it as a way of self-presentation, but downplay this in their reports. People may profit more from self-presentational benefits but construct more favorable motives for their own selfie behavior, in benefit of social demands and their own positive self-view. In this line of interpretation an implication could be that, we need to be aware that selfies are a welcome opportunity to act out self-presentational needs and people even find ways of justification with other hypothetical motivations. In this case, the observed selfie bias may actually fulfill a psychological function. In a way, one may act narcissistic without feeling narcissistic. Beyond this, there are several parallels to described biases and mechanisms in social psychology and self-presentation research which may also help to understand the discrepancy between judgments on own versus others’ selfies.

A first parallel refers to attribution biases. One obvious factor for a more sympathetic interpretation of one’s own selfie behavior may be a classical self-serving bias, i.e., “an ego-biased attribution,” where “we try to explain our behavior in terms that flatter us and put us in a good light” (Miller and Ross, 1975, p. 213). Self-presentational motivations may be associated with narcissism and regarded as less reputable, and therefore attributed to others rather than to oneself. For oneself, one prefers relations to be more reputable character traits such as self-irony or authenticity. This is also in line with previous research on attributions for inconsistencies between online and oﬄine self-presentations (DeAndrea and Walther, 2011). It showed that the types of attributions people made for online behavior depended on the perspective of the person providing the explanation: People explained their own online behavior more favorably than the online behavior of both friends and acquaintances. In short, selfie-takers may protect their self-esteem through claiming socially desirable reasons to take selfies for oneself, instead of less reputable reasons (e.g., narcissistic ambitions) they suspect in others.

Also the fundamental attribution error, i.e., the tendency to focus on internal characteristics (character or intention) in explaining another person’s behavior and situational factors when interpreting one’s own behavior (Jones and Harris, 1967), could play a role for judgments on own versus others’ selfies. While for oneself, one claims that selfies provide authentic insight into real life situations, for others, the inner wish for self-presentation is assigned as more relevant. However, a finding speaking against this interpretation is that people also assigned a higher situational variability to other peoples’ selfies, so that they acknowledge variations from situation to situation. Altogether, the general tendency for self-serving attributions appears as a more obvious factor than the failure to account for situational influences when explaining the behavior of others.

Another relevant factor may be the disregard of bidirectional influences in self-presentational behavior. For example, typical selfie poses, often a bit showy and narcissistic, just become the established way of how to present one self in a selfie and meet our expectations of what a typical selfie looks like. Even if for one self, one may pick the showy pose just “for fun,” does not mean it seriously and rather claims to express self-irony – it is also an invitation for others, to imitate that pose (with the same idea of self-irony), adding to a process of escalating each other’s selfie behavior. People may interpret others’ selfie behavior as mainly driven by self-presentational needs, but underestimate that their own behavior may also have inspired people to such poses. In short, they may neglect, the effects of own self-presentation on self-presentation of others and thus fail to make adequate interpretations of others’ behavior in selfies. This bias has already been described in other contexts. For example, Baumeister et al. (1989) described how people inferred their partners’ self-esteem levels directly from the partners’ behavior, without correcting for how protagonists themselves had altered the partners’ behavior. They then concluded that people may fail to make adequate interpretive adjustments when their self-presentations alter the behavior of others. Again it also shows that people tend to neglect situational influences when evaluating other’s actions.

Though surely not exhausting, the above parallels to popular biases in previous research may help to understand the general importance to understand social media – as inherently social environment – through the lens of social psychology.

## Limitations and Future Research

The present study has several limitations to be addressed in future research. First, the present discussion is only one way of interpretation of correlational results and the overall pattern of findings. This needs to be advanced by (quasi)experimental studies in the future that will allow more accurate interpretations and possibly causal attributions. For example, the described selfie bias is, as most of the described biases in psychology, at first a mere description of systematic shifts between judgments, attributions, or behavior from one context to the other. On the one hand, theoretical analysis, the empirical correlations between habitual self-presentation strategies and selfie-related affect, as well as judgments on others’ selfies suggest their potential for self-presentation to be a prime factor for their wide success. On the other hand, people rather minimize the impact of self-presentation for themselves, and instead, highlight irony and authenticity as more prevalent in their own than others’ selfies. An interesting question for future research would be to gain deeper insight into underlying processes and the relations between these two findings: (1) is there a conscious process underlying? Do people consciously downplay the self-presentational potential of selfies? Do they feel ashamed of their self-presentational needs and try to make up more justified reasons for taking selfies? Or (2) does the observed selfie bias reflect a lack of capability for self-reflection? Do they really perceive their own selfies as more authentic or self-ironic than others’ selfies? Are people not aware of what really attracts them about selfies and may presume other motives for posting selfies than they may actually have? Could the unclear motivation of selfies, open to multiple interpretations, even be a cause for their popularity? Of course, also positions in between are plausible. Future studies could help to get a deeper understanding of the revealed selfie bias and related mechanisms.

Second, our study is based on self-reports and did not include objective data of taking and receiving selfies. We chose this approach due to our main interest in self-reflection and, thus, a lightweight approach to studying the subject. More important than exact information about one selfie more or less was how people perceived their own and others’ selfie behavior and the mental constructions around it. Hence, we aimed to avoid any additional pressure of justification, which might be induced by the study of hard usage data. Along with this, it has to be noted that according to self-reports, our sample was not an overly active sample of selfie-takers, and ambitious selfie-takers with frequencies of several times a week (or more often) formed the minority. Despite this limitation, the found effects are notable, and may be even stronger in a more selfie-focused sample. This, however, has to be validated in future studies, including a higher proportion of heavy selfie takers. In addition, the inclusion of objective usage data could help to advance the present findings and get a more differentiated picture of single phenomena, e.g., the value of sharing selfies versus selfies as a means of documentation for one self. Moreover, methods such as experience sampling (Hektner et al., 2007), a daily diary approach that asks people to report on the nature and quality of their experience related to daily life events, may be adjusted to the context of selfies. Data may be easily collected via smartphone, i.e., the natural object related to taking a selfie. Surveying peoples’ real time-experiences while taking, posting or receiving a selfie will allow deeper insights about which moment actually evokes most positive affect and relevant context factors.

Third, our findings are limited to a European sample, and studying potential intercultural differences for the experience and acceptance of selfies could be an interesting subject for further research. For example, research could contrast individualistic versus collectivistic cultures regarding their selfie culture. One could intuitively assume that selfies, as a highly individual-centered type of photograph may be more accepted in individualistic cultures. On the other hand, especially in many mainly collectivistic Asian countries, placing a high value on interdependence and developing identity through relationship, selfies seem to be quite popular.

It may be that there is another form of interpretation of selfies between different cultures. In our study, most of the participants refused the relation between selfies and relatedness to others and highlighted self-presentation as the most relevant factor. Other cultures may have a different view, and, for example, focus on the collective activity of taking selfies together or posting selfies as an act of creating contact and highlighting togetherness. First hints in that direction can be found in the study by Sung et al. (2016), where communication appeared as main driver of selfie-posting intention, and more individual-centered factors such as attention seeking or narcissism appeared as less relevant. Another aspect could be the high value of social acceptance in collectivist cultures, and liking others’ selfies could be a relevant practice. Instead of an egoist, self-presentational act, the selfie may be interpreted as a sign of appreciation of others’ opinion and asking for confirmation through others.

Forth, future research could examine individual differences that are relevant for the use of self-presentation strategies, and thus, may affect the individual attractiveness of selfies as a self-presentational tool as well. For example, core self evaluation traits (Deci and Ryan, 2002) could play a role, especially the individual autonomy orientation, which reflects a general tendency to base behaviors on core interests and integrated values and to experience true choice in one’s behavior. Given that, people with high autonomy orientation generally make less use of self-presentation strategies (Lewis and Neighbors, 2005), a high autonomy orientation may also diminish the interest in selfies or other forms of self-presentation in social media.

Finally, our study of relations between selfies and habitual self-presentation strategies was limited to a particular set of self-presentation strategies. Aiming for a parsimonious research design, which focused on those strategies we assumed as most fitting or non-fitting for selfies. However, future research could include further self-presentation strategies. This could also include the study of relations to different motivations behind self-presentation. For example, a prominent distinction of self-presentational motivations is self-construction/self-fulfillment versus obtaining rewards from others, and, thus, pleasing the audience (Baumeister, 1982). This distinction also shows parallels to different researchers’ positions on the value of selfies, such as that selfies are a means for self and identity exploration (Rutledge, 2013), selfies as a practice of freedom, or self-therapeutic and awareness-raising practice (Tiidenberg and Cruz, 2015) in contrast to others promoting the impression management motivation and the fabrication of selfies to disseminate desired impressions to others (Lyu, 2016).

## Conclusion

As the present study showed, self-presentation may be a central factor for the attractiveness of selfies but at the same time is downplayed in self-reports. While many people are contributing to the success of selfies, only few declare true commitment. In the end, however, the combination of these two factors, an opportunity for self-presentation without an obvious revelation of self-presentational needs, may also be part of the secret of their success. What we here called the selfie paradox and selfie bias could also be a key factor for their popularity. Forming a lightweight possibility for self-presentation, that allows people to strategically adjust and experiment with the impression they make on others, but still in a playful and somewhat ambiguous manner, that is even interpreted as self-irony (at least by the selfie-takers themselves).

Clever experimental studies will surely shed further light on the exact motivations behind selfies. But in daily life, one’s specific motivations for taking a selfie usually remain uncovered. Others, and possibly even oneself, can never have full and final insight into what motivates taking a selfie, and this might actually be what attracts people. In this sense, the present research also adds to a deeper understanding of success factors for social media in general. In the end it might be all about fulfilling basic human needs (here: popularity, self-expression) in a way that feels good for people, does not reveal too much about deeper motivations and allows them to keep a positive self-view and image to others.

## Ethics Statement

An ethics committee approval was not requested. The study was conducted via online survey and did not include any experimental manipulation or deception about the study’s purpose. Participants were free to stop participation at any time.

## Author Contributions

SD and LC designed and conducted the research. SD analyzed the data and drafted the manuscript, LC critically revised the manuscript. Both authors approved the final version of the manuscript for submission.

## Conflict of Interest Statement

The authors declare that the research was conducted in the absence of any commercial or financial relationships that could be construed as a potential conflict of interest.

## Footnotes

1 http://i2.kym-cdn.com/photos/images/original/000/518/574/17c.jpg

2 http://i0.kym-cdn.com/photos/images/original/000/604/134/e4b.jpg

3 unipark.com
